# Supplementary material for: Comparing Machine Learning and Nurse Predictions for Hospital Admissions in a Multisite Emergency Care System
Source: medRxiv. 2025 Apr 8:2025.04.07.25325126. Preprint. [Version 1] doi: 10.1101/2025.04.07.25325126 (PMC12036418; doi:10.1101/2025.04.07.25325126)
Supplement: Supplement 1 [file media-1.pdf]

Supplementary Content

Table S1. Ten most common chief complaints among patients by admission status, ranked by frequency............2

Table S2. Most common past medical history CCS categories among discharged and admitted patients...............2

Machine Learning Models Hyper-Parameters Tuning..4-13

Table S3. XGBoost model performance evaluated across different hyperparameter settings.................................4

Figure S1. Single feature analysis in the XGBoost model..6

Tables S4-5. Bio-clinical-BERT hyperparameter tuning results...7-11

Table S6. performance metrics of the XGBoost model at different probability thresholds....................................12

Table S7. Performance metrics of the NLP model at different probability thresholds...13

Rank	Chief Complaint (Discharged)	Frequency (n=1,501,012)	%	Chief Complaint (Admitted)	Frequency (n=319,294)	%
1	Unspecified	197,400	10.9%	Unspecified	39,703	9.9%
2	Abdominal Pain	142,844	7.9%	Shortness Of Breath	37,210	9.3%
3	Other	129,440	7.1%	Abdominal Pain	37,142	9.3%
4	Chest Pain	80,442	4.4%	Other	28,533	7.1%
5	Cough	61,590	3.4%	Chest Pain	21,271	5.3%
6	Fever	60,358	3.3%	Fall	14,497	3.6%
7	Back Pain	53,730	3.0%	Weakness	14,103	3.5%
8	Headache	52,418	2.9%	Fever	14,046	3.5%
9	Shortness Of Breath	52,220	2.9%	Vomiting	13,541	3.4%
10	Vomiting	50,461	2.8%	Altered Mental Status	12,192	3.0%

Supplementary Table S1: the ten most common chief complaints among patients by admission status, ranked by frequency.

Rank	CCS Classification	Frequency (Discharged, n=1,501,012)	CCS Classification	Frequency (Admitted, n=319,294)

1	FAC (Factors Influencing Health Status)	3,270,544	SYM (Symptoms, Signs, Abnormal Findings)	1,160,765
2	SYM (Symptoms, Signs, Abnormal Findings)	3,246,235	CIR (Diseases of the Circulatory System)	1,082,625
3	MUS (Diseases of the Musculoskeletal System and Connective Tissue)	1,856,176	FAC (Factors Influencing Health Status)	999,920
4	CIR (Diseases of the Circulatory System)	1,830,674	END (Endocrine, Nutritional, and Metabolic Diseases)	660,738
5	INJ (Injury, Poisoning, and Other External Causes)	1,504,222	MUS (Diseases of the Musculoskeletal System and Connective Tissue)	632,383
6	GEN (Diseases of the Genitourinary System)	1,338,750	DIG (Diseases of the Digestive System)	506,380
7	END (Endocrine, Nutritional, and Metabolic Diseases)	1,336,579	GEN (Diseases of the Genitourinary System)	504,874
8	MBD (Mental, Behavioral, and Neurodevelopmental Disorders)	1,280,195	INJ (Injury, Poisoning, and Other External Causes)	483,500
9	DIG (Diseases of the Digestive System)	1,275,152	RSP (Diseases of the Respiratory System)	405,380
10	RSP (Diseases of the Respiratory System)	1,199,335	NVS (Diseases of the Nervous System)	372,728

Supplementary Table S2: most common past medical history CCS categories among discharged and admitted patients.

Machine Learning Models Hyper-Parameters Tuning

XGBoost Model Performance and Hyperparameter Tuning

Hyperparameter tuning for the XGBoost model showed that admission prediction performance, measured by AUC, improved with specific configurations (**Supplementary Table S3**). The highest AUC score of 0.871 was achieved with n_estimators=1000, learning_rate=0.1, and max_depth=6, indicating this as the optimal setting. Performance generally increased with greater n_estimators and max_depth, particularly when paired with a moderate learning rate, reflecting the model's sensitivity to deeper trees and a balanced learning pace.

**n_estimators**	**learning_rate**	**max_depth**	**AUC Score**
300	0.05	3	0.853
300	0.05	5	0.860
300	0.05	6	0.862
300	0.1	3	0.859
300	0.1	5	0.865
300	0.1	6	0.866
300	0.3	3	0.865
300	0.3	5	0.868
300	0.3	6	0.868

500	0.05	3	0.857
500	0.05	5	0.864
500	0.05	6	0.865
500	0.1	3	0.863
500	0.1	5	0.867
500	0.1	6	0.869
500	0.3	3	0.867
500	0.3	5	0.869
500	0.3	6	0.869
1000	0.05	3	0.862
1000	0.05	5	0.867
1000	0.05	6	0.869
1000	0.1	3	0.866
1000	0.1	5	0.870
**1000**	**0.1**	**6**	**0.871**
1000	0.3	3	0.869
1000	0.3	5	0.869
1000	0.3	6	0.867

Supplementary Table S3: The table displays the AUC scores for XGBoost model performance on test data, evaluated across different hyperparameter settings. Each row represents a unique combination of n_estimators, learning_rate, and max_depth.

Single Feature Analysis

Single feature analysis showed that "CCS set" (paste medical history), "Age," and "ESI" had the top three highest predictive power, with AUC scores of 0.773, 0.726, and 0.707, respectively. In contrast, features like "Sex" and "Respirations" showed minimal predictive value, emphasizing the varying strength of individual features in admission prediction (**Supplementary Figure S1**).

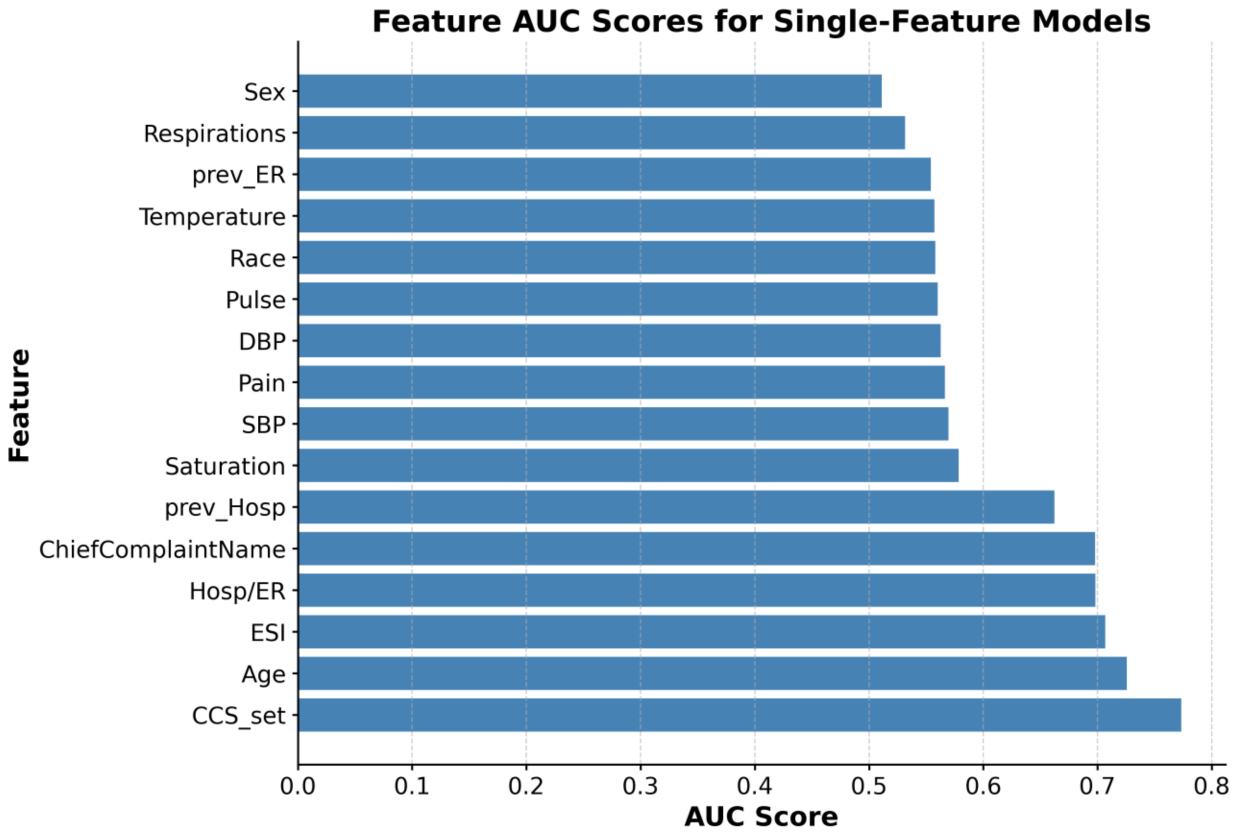

Supplementary Figure S1: This figure displays the AUC scores for individual features in the XGBoost model, highlighting each feature's standalone predictive power for admission.

Hyperparameter Tuning for Bio-clinical-BERT: Initial Exploration

We explored Bio-clinical-BERT performance with different learning rates (lr), batch sizes (bs), and weight decay (wd) using a random set of 10k samples from the training set and a random set of 2k samples from the internal validation set. Performance was evaluated using AUC. The highest AUC achieved was 0.819 with a learning rate of 10^{-5}, batch size of 8, and weight decay of 0.01 (**Supplementary Table S4**).

**Learning Rate (lr)**	**Batch Size (bs)**	**Weight Decay (wd)**	**AUC**
0.00001	8	0.01	0.819
0.00005	128	0.01	0.819
0.00005	64	0.01	0.818
0.00002	16	0.10	0.818
0.00002	32	0.01	0.818
0.00001	16	0.01	0.817
0.00005	128	0.10	0.817
0.00001	8	0.10	0.816
0.00002	8	0.10	0.816
0.00002	16	0.01	0.816

0.00002	64	0.10	0.815
0.00002	8	0.01	0.815
0.00005	16	0.01	0.815
0.00005	32	0.10	0.813
0.00001	32	0.10	0.812
0.00005	16	0.10	0.812
0.00001	16	0.10	0.811
0.00002	64	0.01	0.810
0.00001	32	0.01	0.809
0.00005	32	0.01	0.809
0.00005	8	0.10	0.806
0.00005	64	0.10	0.806
0.00005	256	0.01	0.801
0.00005	256	0.10	0.800
0.00002	128	0.10	0.800
0.00001	64	0.01	0.800
0.00005	8	0.01	0.799
0.00001	64	0.10	0.793

0.00002	128	0.01	0.792
0.00002	256	0.01	0.777
0.00002	256	0.10	0.772
0.00001	128	0.01	0.753
0.00001	128	0.10	0.746
0.00001	256	0.01	0.706
0.00001	256	0.10	0.697
0.00005	32	0.10	0.478

Supplementary Table S4: Bio-clinical-BERT hyperparameter tuning results.

The model was further evaluated with an expanded dataset of 50k training samples and 10k test samples. The highest AUC achieved was 0.840 with a learning rate 10^{-5}, batch size of 8, and weight decay of 0.01 (**Supplementary Table S5**).

Given the operational constraints and efficiency considerations, we selected the top-performing configuration with a batch size of 256 for the final model. This configuration achieved an AUC of 0.838 with a learning rate of $5x10^{-6}$ and weight decay of 0.10, balancing strong performance with computational scalability.

Learning Rate (lr)	Batch Size (bs)	Weight Decay (wd)	AUC

0.00001	8	0.01	0.840
0.00002	32	0.01	0.838
0.00002	64	0.01	0.838
0.00005	256	0.10	0.838
0.00002	16	0.01	0.838
0.00002	8	0.01	0.837
0.00001	16	0.01	0.837
0.00001	16	0.10	0.837
0.00005	64	0.01	0.836
0.00002	32	0.10	0.836
0.00001	32	0.10	0.836
0.00002	128	0.10	0.836
0.00001	8	0.10	0.836
0.00001	64	0.10	0.836
0.00001	64	0.01	0.836
0.00002	128	0.01	0.835
0.00005	256	0.01	0.835
0.00002	16	0.10	0.835

0.00005	128	0.10	0.835
0.00005	32	0.01	0.834
0.00002	8	0.10	0.834
0.00005	16	0.01	0.834
0.00001	32	0.01	0.834
0.00005	64	0.10	0.834
0.00002	64	0.10	0.834
0.00002	256	0.01	0.834
0.00002	256	0.10	0.833
0.00005	128	0.01	0.832
0.00005	16	0.10	0.832
0.00001	128	0.10	0.832
0.00005	8	0.01	0.831
0.00005	8	0.10	0.829
0.00001	128	0.01	0.829
0.00001	256	0.10	0.824
0.00002	32	0.10	0.823
0.00001	256	0.01	0.823

Supplementary Table S5: Hyperparameter tuning results (training set of 50,000 visits).

Threshold	Accuracy	F1 Score	Sensitivity	Specificity	NPV	PPV
0.10	69.4% (69.0% - 69.9%)	0.53 (0.52 - 0.54)	90.0% (89.4% - 90.6%)	64.5% (64.0% - 65.0%)	96.4% (96.2% - 96.7%)	37.7% (37.1% - 38.4%)
0.19 (Youden's index)	79.7% (79.4% - 80.1%)	0.60 (0.59 - 0.61)	78.9% (78.1% - 79.8%)	79.9% (79.5% - 80.4%)	94.1% (93.8% - 94.3%)	48.4% (47.7% - 49.2%)
0.30	84.2% (83.9% - 84.6%)	0.62 (0.61 - 0.63)	66.8% (65.9% - 67.8%)	88.4% (88.1% - 88.7%)	91.8% (91.5% - 92.1%)	57.9% (56.9% - 58.8%)
0.50	85.9% (85.5% - 86.2%)	0.55 (0.54 - 0.56)	45.3% (44.3% - 46.4%)	95.6% (95.4% - 95.8%)	88.0% (87.6% - 88.3%)	70.9% (69.7% - 72.0%)
0.70	84.3% (84.0% - 84.7%)	0.37 (0.36 - 0.38)	23.6% (22.8% - 24.5%)	98.8% (98.7% - 98.9%)	84.4% (84.1% - 84.8%)	82.6% (81.0% - 84.1%)
0.90	81.7% (81.4% - 82.1%)	0.10 (0.10 - 0.11)	5.5% (5.0% - 6.0%)	99.9% (99.9% - 99.9%)	81.6% (81.2% - 81.9%)	94.1% (91.9% - 96.0%)

Supplementary Table S6: performance metrics of the XGBoost model at different probability thresholds. Metrics include F1, accuracy, sensitivity, specificity, and predictive values with 95% confidence intervals.

Threshold	Accuracy	F1 Score	Sensitivity	Specificity	NPV	PPV
0.10	64.2% (63.7% - 64.6%)	0.50 (0.49 - 0.50)	90.7% (90.1% - 91.3%)	57.7% (57.2% - 58.2%)	96.3% (96.0% - 96.5%)	34.1% (33.5% - 34.7%)

0.19 (Youden's index)	76.4% (76.1% - 76.8%)	0.57 (0.56 - 0.58)	79.7% (78.8% - 80.5%)	75.7% (75.2% - 76.1%)	93.9% (93.6% - 94.2%)	44.2% (43.4% - 44.9%)
0.30	83.1% (82.8% - 83.4%)	0.60 (0.59 - 0.61)	65.4% (64.4% - 66.3%)	87.4% (87.1% - 87.7%)	91.3% (91.0% - 91.6%)	55.6% (54.6% - 56.5%)
0.50	85.6% (85.3% - 85.9%)	0.55 (0.54 - 0.56)	45.1% (44.1% - 46.2%)	95.4% (95.2% - 95.6%)	87.8% (87.5% - 88.1%)	70.2% (69.0% - 71.4%)
0.70	84.6% (84.3% - 84.9%)	0.40 (0.39 - 0.41)	26.7% (25.8% - 27.6%)	98.6% (98.5% - 98.7%)	84.8% (84.5% - 85.1%)	82.3% (80.9% - 83.7%)
0.90	82.4% (82.1% - 82.8%)	0.19 (0.17 - 0.20)	10.3% (9.6% - 10.9%)	99.8% (99.8% - 99.9%)	82.2% (81.8% - 82.5%)	93.0% (91.4% - 94.5%)

Supplementary Table S7: Performance metrics of the NLP model at different probability thresholds. Metrics include F1, accuracy, sensitivity, specificity, and predictive values with 95% confidence intervals.
